# Etravirine as a Switching Option for Patients with HIV RNA Suppression: A Review of Recent Trials

**DOI:** 10.1155/2014/636584

**Published:** 2014-02-25

**Authors:** Mark Nelson, Andrew Hill, Yvon van Delft, Christiane Moecklinghoff

**Affiliations:** ^1^Chelsea and Westminster Hospital, St. Stephens Centre, London SW10 9NH, UK; ^2^Janssen Research and Development, High Wycombe HP12 4DP, UK; ^3^Janssen EMEA, Tilburg, The Netherlands; ^4^Janssen EMEA, Neuss, Germany

## Abstract

Unlike other nonnucleoside reverse transcriptase inhibitors, etravirine is only approved for use in treatment-experienced patients. In the DUET 1 and 2 trials, 1203 highly treatment-experienced patients were randomized to etravirine or placebo, in combination with darunavir/ritonavir and optimized background treatment. In these trials, etravirine showed significantly higher rates of HIV RNA suppression when compared with placebo (61% versus 40% at Week 48). There was no significant rise of lipids or neuropsychiatric adverse events, but there was an increase in the risk of rash with etravirine treatment. In the SENSE trial, which evaluated etravirine and efavirenz in 157 treatment-naïve patients in combination with 2 nucleoside analogues, there was a lower risk of lipid elevations and neuropsychiatric adverse events with etravirine when compared to efavirenz. Etravirine has been evaluated in three randomized switching studies. In the SSAT029 switch trial, 38 patients who had neuropsychiatric adverse events possibly related to efavirenz showed an improvement in these after switching to etravirine. The Swiss Switch-EE recruited 58 individuals without neuropsychiatric adverse events who were receiving efavirenz, and no benefit was shown when switching to etravirine. In the Spanish ETRA-SWITCH trial (*n* = 46), there were improvements in lipids when individuals switched from a protease inhibitor to etravirine. These switching trials were conducted in patients with full HIV RNA suppression: <50 copies/mL and with no history of virological failure or resistance to therapy. The results from these three randomized switching studies suggest a possible new role for etravirine, in combination with two nucleoside analogues, as a switching option for those with HIV RNA suppression but who are reporting adverse events possibly related to antiretroviral therapy. However a large well-powered trial would need to be conducted to strengthen the evidence from the pilot studies conducted so far.

## 1. Introduction

There are four nonnucleoside analogues recommended in Europe and North America for the treatment of HIV: efavirenz, etravirine, nevirapine, and rilpivirine [1–3]. Of these, efavirenz is the most widely recommended for first-line treatment, owing to the high rates of efficacy seen in large randomized trials [4–6]. Nevirapine has shown levels of efficacy close to, but not equivalent with, efavirenz as a first-line treatment [7]. Nevirapine has recently been reformulated to a 400 mg once daily extended release (XR) dosing, which has shown noninferior efficacy to the original dose of 200 mg twice daily in treatment-naïve patients [8]. Rilpivirine showed improved tolerability versus efavirenz in the ECHO and THRIVE studies, but higher rates of virological failure among those with baseline HIV RNA levels above 100,000 copies/mL [9].

Efavirenz and rilpivirine have both been coformulated with tenofovir and emtricitabine into a single pill which is prescribed once daily: Atripla for efavirenz and Complera/Eviplera for rilpivirine [10, 11]. Nevirapine XR has been developed as a single pill for once daily first-line use but is combined with two nucleoside analogues which are prescribed separately from the nevirapine [8]. Etravirine has only been approved for treatment of experienced patients [12]. Etravirine has not been coformulated with nucleoside analogues, and the daily approved dose involves two 200 mg tablets [13]. Despite these possible drawbacks, etravirine may have a role as a switch option for patients who have HIV RNA suppression on other treatments but have developed adverse events.

There are several concerns over the safety profiles of efavirenz and nevirapine. Efavirenz causes a range of neuropsychiatric adverse events, in particular dizziness and mood and sleep disorders [14, 15]. These adverse events are generally mild and short term, but in some may be long-lasting. Efavirenz also causes rises in lipids [16] and there is a risk of rash [17]. Efavirenz showed teratogenicity in animal models, but the most recent guidance from the World Health Organization and the British HIV Association does permit continued use of efavirenz in pregnancy [18, 19]. This is because of evidence from a meta-analysis of outcomes in pregnant women treated with efavirenz showing no excess of birth abnormalities [20]. Nevirapine has been associated with severe skin reactions in a minority of patients, including Stevens-Johnsons syndrome, particularly if used in patients with high CD4 counts [7]; hepatotoxicity is an additional issue [21].

Patients with virological failure while taking first-line efavirenz, nevirapine, or rilpivirine have a significant risk of developing resistance to nonnucleosides and nucleoside analogues, which can restrict future treatment options [22, 23]. However etravirine has been shown to have antiviral activity against HIV with resistance to efavirenz or nevirapine [24, 25].

In this review, we discuss the potential role for etravirine as a switch option in those who develop adverse events while taking alternate antiretroviral treatments. Efficacy and safety results from the original DUET trials in highly experienced patients are discussed, together with the SENSE trial in first-line treatment. Three randomized studies evaluating the switch from efavirenz to etravirine have been conducted and are reviewed. The designs of these trials are shown in Table 1.

## 2. The Development of Etravirine

The nonnucleoside etravirine has *in vitro* activity against both NNRTI-naïve and resistant viruses [24, 25]. A proof-of-concept trial showed significant reductions in HIV RNA in treatment-naïve individuals receiving etravirine as monotherapy for seven days [32]. A pilot study of etravirine was conducted (TMC125-C227) in NRTI and NNRTI experienced patients, comparing second-line treatment with optimized nucleoside analogues plus either etravirine or protease inhibitors [33]. This study was discontinued early, owing to a higher rate of virological failure in the etravirine arm. The results revealed that etravirine is needed to be used in combination with fully active antiretrovirals to ensure HIV RNA suppression.

Unlike other nonnucleoside analogues, etravirine was first evaluated in highly treatment-experienced patients. The results from the DUET 1 and 2 trials, where etravirine was evaluated in combination with darunavir/ritonavir, led to the regulatory approval for treatment-experienced patients at a 200 mg twice daily dose [26, 27].

Although etravirine is approved at the 200 mg twice daily dose, the long half-life of etravirine (30–40 hours) supports once daily dosing [34]. Pharmacokinetic studies have evaluated the 400 mg once daily dose of etravirine in treatment-naïve patients [35] and those switching from efavirenz [36] and those switching from the 200 mg twice daily dose of etravirine [37]. The results from these pharmacokinetic studies led to the once daily dose used in the SENSE trial and the switching studies described below.

After the completion of the DUET trials, extended pharmacovigilance demonstrated three cases of severe rash or hypersensitivity during etravirine treatment in routine clinical practice [12].

## 3. The DUET Trials

The DUET 1 and 2 trials recruited 1203 patients with preexisting resistance to nonnucleoside and protease inhibitors [26, 27]. The patients were randomized to either etravirine or placebo, which were prescribed with a background regimen (BR) of darunavir/ritonavir, nucleoside analogues, and optional enfuvirtide. This was a double-blind, placebo-controlled trial. Summary results from the DUET trials at Week 48 are shown in Table 2. Despite the high prevalence of NNRTI resistance at baseline, there was an efficacy advantage for the etravirine arm over placebo: 61% of patients having HIV RNA suppression below 50 copies/mL at Week 48 and 40% in the placebo arm. These efficacy benefits were maintained at Week 96 [27]. There was no increase in the risk of nervous system or psychiatric adverse events or in lipids in the etravirine arm when compared with placebo although there was a higher risk of Grades 1–4 rash with etravirine (10% versus 4%). However the number of patients with serious rash (Grade 3 or 4) was 1.3% with etravirine and 0% with placebo; 2.2% of patients discontinued the etravirine arm due to rash and 0% with placebo. There was no significant increase in the risk of lipid abnormalities with etravirine when compared to placebo (Table 1).

Since the DUET trials were completed, etravirine has been combined with other novel antiretrovirals in triple combinations for use in highly treatment-experienced patients, such as darunavir/ritonavir and raltegravir [38, 39].

## 4. The SENSE Trial

The double-blind, placebo-controlled SENSE trial [28] evaluated the efficacy and safety of etravirine and efavirenz in individuals naïve to antiretroviral therapy. Patients with HIV RNA >5000 copies/mL were randomised to etravirine 400 mg once daily (*n* = 79) or efavirenz 600 mg (*n* = 78), plus two nucleoside analogues. The primary objective of the trial was to compare the risk of neuropsychiatric adverse events after 12 weeks of treatment. However, efficacy was also evaluated, and patients were followed up on randomised treatment to Week 48. Summary results are shown in Table 3.

In the Intent to Treat analysis at Week 48, 60/79 (76%) on etravirine and 58/78 (74%) on efavirenz had a HIV RNA <50 copies/mL. In the on Treatment analysis, there were 92% with HIV RNA <50 copies/mL for etravirine and 89% for efavirenz. Etravirine showed noninferior efficacy versus efavirenz in both analyses (*P* < 0.05) [28]. Four patients had virological failure in the etravirine arm; none developed resistance to nucleoside analogues or nonnucleosides. Seven demonstrated virological failure in the efavirenz arm; three developed treatment-emergent resistance to nucleoside analogues and/or nonnucleosides.

There were safety benefits to etravirine over efavirenz in this trial (Table 3). The number of patients who developed Grades 2–4 drug-related adverse events was smaller with etravirine. Fewer individuals discontinued from the etravirine arm for adverse events. The risk of nervous system or psychiatric adverse events was lower with etravirine. This difference persisted throughout the trial; at Week 48 visit, the percentage with ongoing neuropsychiatric adverse events was 6.3% for etravirine and 21.5% for efavirenz [28]. Fewer patients developed elevations in lipids in the etravirine arm. However the risk of developing skin or subcutaneous disorders was similar with the two drugs.

The SENSE trial was not designed to demonstrate differences in efficacy for etravirine when compared with efavirenz in first-line treatment, and etravirine is still only approved for use in treatment-experienced patients. However the results from the trial do highlight the differences in safety and tolerability between etravirine and efavirenz.

## 5. The UK SSAT029 Trial

Results from an open-label pilot study suggested that patients with undetectable HIV RNA could be successfully switched to etravirine [40]. Consequently, a double-blind, placebo-controlled cross over trial was designed to evaluate whether a switch from efavirenz to etravirine could lead to improvements in nervous system and psychiatric side effects [29]. The trial recruited 38 patients who were receiving efavirenz with two nucleoside analogues and who had HIV RNA suppression <50 copies/mL, but ongoing CNS symptoms possibly related to efavirenz. In the immediate switch arm, patients changed from efavirenz to etravirine 400 mg once daily for 24 weeks, while continuing their nucleoside analogues. In the delayed switch arm, patients maintained their pretrial treatment for 12 weeks, and then switched to etravirine at Week 12, continuing to Week 24. Patients were evaluated for adverse events after 12 weeks and 24 weeks of treatment.

At Week 12 visit there was a significant reduction in CNS adverse events in the patients in the immediate switch arm, whereas there was no change in CNS symptoms in the patients continuing efavirenz (Table 4). The patients in the delayed arm who then switched to etravirine at Week 12 showed improvements in their CNS adverse events by Week 24. Table 4 shows the overall improvements in CNS adverse events after switching to etravirine, combining both treatment arms. Switching from efavirenz to etravirine led to statistically significant improvements in overall adverse events, insomnia, abnormal dreams, and nervousness (*P* < 0.05 for each comparison). In addition there were statistically significant reductions in total cholesterol after the switch from efavirenz to etravirine. In the analysis of clinical adverse events, there was no difference between the arms for depression (Table 4). All patients maintained HIV RNA levels below 50 copies/mL at all study visits.

## 6. Swiss SWITCH-EE Trial

This was a double-blind, placebo-controlled cross over trial, evaluating 58 individuals who had no side effects and had tolerated efavirenz for at least three months with HIV RNA suppression below 50 copies/mL [30]. In a cross over design, the patients received either etravirine 400 mg once daily or efavirenz 600 mg once daily plus 2 NRTIs for six weeks, and then switched to the alternate NNRTI for a further six weeks. The primary endpoint was patient preference for the first or second NNRTI received and was assessed after the full 12 weeks of the trial.

At the end of the trial, there was no significant difference in preference for either NNRTI. However there was an association between patient preference and the order in which patients received the NNRTIs: patients who continued efavirenz during the first phase of the trial preferred efavirenz (15/21 = 71%), whereas those who commenced with etravirine in the first phase preferred etravirine (16/17 = 94%). Quality of sleep, depression, anxiety, and stress scores did not differ significantly between the two groups. The median lipid levels improved during the etravirine phase of the trial (Table 5).

The main safety results from this trial differ from the UK SSAT029 trial, described above. In the Swiss SWITCH-EE trial, patients did not have possible efavirenz related neuropsychiatric adverse events at baseline, whereas this was an entry requirement for the UK SSAT029 study. In addition, the authors of the Swiss SWITCH-EE study suggested that the order effect of preference in this trial could be related to the reemergence of neuropsychiatric adverse events in the group who switched to etravirine during the first phase and then restarted efavirenz in the second phase. By contrast, the patients who continued efavirenz for the first six weeks, and then switched to etravirine later, would not have experienced reemergence of these neuropsychiatric adverse events. All patients maintained HIV RNA levels below 50 copies/mL during the 12-week trial.

## 7. Spanish ETRA-Switch Trial

This 48-week trial recruited 46 patients with HIV RNA suppression <50 copies/mL while receiving PI-based treatment [31]. Patients recruited had a history of either dyslipidemia, gastrointestinal disturbancem or low satisfaction with their current antiretroviral regimen. Patients with a history of NRTI or NNRTI resistance were excluded from the trial. The patients were randomized to either continue their protease inhibitor or switch to etravirine 400 mg once daily for 48 weeks.

The 24-week results from this study have been presented. All patients maintained HIV RNA levels below 50 copies/mL to Week 24. There were statistically significant reductions in total cholesterol, HDL cholesterol, and triglycerides in the etravirine arm (Table 5) with no significant change in the protease inhibitor arm. One patient in each arm discontinued from the trial for adverse events (gastrointestinal disturbance in the etravirine arm and hypercholesterolemia in the protease inhibitor arm). In a pharmacokinetic substudy, those in the etravirine arm maintained etravirine trough concentrations above 4 ng/mL throughout the trial.

## 8. Etravirine in Novel Combinations

In addition to the switch studies described above, etravirine has been evaluated in pilot studies in combination with other new antiretrovirals [41–43]. For patients who already have resistance to nucleoside analogues, nonnucleosides, and protease inhibitors, the combination of etravirine with the integrase inhibitor raltegravir, the CCR5 antagonist maraviroc, or the protease inhibitor darunavir/ritonavir could provide a high enough genetic barrier to overcome high levels of preexisting drug resistance.

## 9. Conclusions

The combined results from the randomised trials of etravirine suggest a role as a switch option for patients who have adverse events while receiving other antiretroviral treatments. Patients who have ongoing neuropsychiatric adverse events while receiving efavirenz show improvements after switching to etravirine based on the UK SSAT029 study [29]. However in the Swiss SWITCH-EE study, where individuals did not report neuropsychiatric adverse events at screening, there was no significant benefit from the switch to etravirine [30]. The results from the Spanish ETRA-SWITCH trial show improvements in lipids after switching from a protease inhibitor to etravirine [31]. There were also improvements in lipids with etravirine when compared to efavirenz in the SENSE, Swiss SWITCH-EE, and UK SSAT029 trials. The clinical significance of these changes in lipids is unclear.

The clinical trials evaluating these switches were not statistically powered to demonstrate equivalent rates of HIV RNA suppression for etravirine when compared with the control arm [44]. Patients should be carefully selected on the basis of the inclusion criteria for the trials, including HIV RNA suppression <50 copies/mL and no evidence for NRTI/NNRTI resistance during prior virological failure. A new randomised clinical trial, powered for noninferiority would be required to validate the results from the pilot studies conducted so far. This trial would need to show that a switch to etravirine maintained HIV RNA suppression in people previously suppressed on other treatments and also provided a safety benefit.

## Figures and Tables

**Table 1:** Design of the six main randomized trials of etravirine.

Trial [ref]	Treatment arms (*n*)	Inclusion	Duration	Primary endpoint
DUET [26, 27]	BR + ETR (*n* = 599)	Experienced	96 weeks	HIV RNA <50 c/mL
	BR + placebo (*n* = 604)			

SENSE [28]	2NRTI + ETR (*n* = 77)	Naïve	48 weeks	CNS adverse events
	2NRTI + EFV (*n* = 79)			

UK SSAT029 [29]	2NRTI + ETR (*n* = 20)	HIV RNA <50	24 weeks	CNS adverse events
	2NRTI + EFV (*n* = 18)			

Swiss SWITCH-EE [30]	2NRTI + ETR (*n* = 28)	HIV RNA <50	12 weeks	Patient satisfaction
	2NRTI + EFV (*n* = 30)			

ETRA-SWITCH [31]	2NRTI + ETR (*n* = 24)	HIV RNA <50	48 weeks	HIV RNA <50 c/mL
	2NRTI + PI/r (*n* = 22)			

**Table 2:** DUET trials: summary efficacy and safety results by treatment arm, to Week 48.

	Etravirine + BR (*n* = 599)	Placebo + BR (*n* = 604)
HIV RNA <50 c/mL, ITT	363 (61%)	240 (40%)
Any grade 3 or 4 adverse event	199 (33%)	211 (35%)
Adverse events leading to discontinuation	43 (7%)	34 (6%)
Grades 1–4 clinical adverse events of interest		
Nervous system	103 (17%)	119 (20%)
Psychiatric	100 (17%)	118 (20%)
Rash	60 (10%)	21 (4%)*
Grades 3-4 laboratory abnormalities		
Elevated ALT	22 (4%)	12 (2%)
Elevated AST	19 (3%)	12 (2%)
Elevated total cholesterol	48 (8%)	32 (5%)
Elevated LDL	42 (7%)	39 (7%)
Elevated triglycerides	55 (10%)	35 (6%)

BR: background regimen of optimized nucleoside analogues plus optional enfuvirtide.

*P < 0.05, comparison between treatment arms at Week 48.

**Table 3:** SENSE trial: summary efficacy and safety results by treatment arm, to Week 48.

	Etravirine arm (*n* = 79)	Efavirenz arm (*n* = 78)
HIV RNA <50 c/mL, ITT	60 (76%)	58 (74%)
Any Grades 2–4 drug-related adverse event	21 (27%)	33 (42%)*
Adverse events leading to discontinuation	6 (8%)	13 (17%)*
Grades 2–4 drug-related clinical adverse events of interest		
Nervous system	1 (1%)	13 (17%)*
Psychiatric	4 (5%)	12 (15%)*
Skin or subcutaneous disorders	9 (11.4%)	9 (11.5%)
Grades 3-4 laboratory abnormalities		
Hypophosphatemia	0	4 (5%)
Neutropenia	6 (8%)	3 (4%)
Elevated ALT	2 (3%)	1 (1%)
Elevated AST	1 (1%)	2 (3%)
Elevated total cholesterol	1 (1%)	6 (8%)
Elevated LDL	2 (3%)	8 (10%)
Elevated triglycerides	0	2 (3%)

*P < 0.05, comparison between treatment arms at Week 48.

**Table 4:** UK SSAT029 trial: changes in CNS adverse events by treatment arm, to Week 12.

	Etravirine*(*n* = 20)	Efavirenz*(*n* = 18)
Grades 2–4 CNS adverse events		
All Grades 2–4 CNS adverse events	60%	81%
Median number of CNS adverse events	1	3
Dizziness	15%	19%
Depression	20%	19%
Insomnia	37%	60%
Anxiety	25%	44%
Impaired concentration	30%	31%
Headache	5%	25%
Somnolence	30%	31%
Fatigue	35%	44%
Abnormal dreams	20%	63%
Nervousness	9%	29%
Hallucinations	0%	7%

*Patients in the delayed (efavirenz) arm switched to etravirine at Week 12; patients in the immediate etravirine arm remained on etravirine after Week 12.

**Table:** (a) Swiss SWITCH-EE trial: changes in lipids six weeks after switching from efavirenz to etravirine treatment (mmol/L)

Lab parameter Median (IQ range)	End of efavirenz phase	End of etravirine phase	Change between EFV and ETR phases (*n* = 55)
ALT	33.0 (24–55.5)	37.0 (26–64)	0.0 (−5.5, 6.0)
Total cholesterol	5.5 (4.7–6.3)	4.6 (3.9–5.5)	−0.7 (−1.1, −0.2)*
HDL cholesterol	1.1 (0.9–1.4)	1.1 (0.9–1.3)	−0.02 (−0.1, +0.1)
LDL cholesterol	3.3 (2.6–3.8)	2.8 (2.1–3.3)	−0.6 (−0.7, −0.1)*
Triglycerides	1.7 (1.2–2.4)	1.4 (1.0–1.9)	−0.3 (−0.9, −0.1)*

**Table:** (b) ETRA-SWITCH trial: changes in lipids from baseline, to Week 24 (mmol/L)

Mean lipid levels (95% C.I)	Etravirine group		PI control group	
	Baseline	Week 24	Baseline	Week 24
Total cholesterol	5.33 (4.48–6.31)	4.81 (4.14–5.64)*	4.82 (4.30–5.70)	5.39 (4.51–6.01)
HDL-cholesterol	1.35 (1.01–1.66)	1.24 (0.88–1.61)*	1.30 (1.04–1.58)	1.24 (1.01–1.37)
LDL cholesterol	2.85 (2.41–3.83)	3.13 (2.36–3.52)	2.98 (2.43–3.52)	2.77 (2.20–3.68)
Triglycerides	1.89 (1.20–2.60)	1.21 (0.90–1.50)*	1.20 (0.80–2.01)	1.40 (0.85–2.73)

*P < 0.05, comparison between treatments; IQ range: interquartile range; ETR: etravirine; EFV: efavirenz.
